# ZNF674-AS1 antagonizes miR-423-3p to induce G0/G1 cell cycle arrest in non-small cell lung cancer cells

**DOI:** 10.1186/s11658-021-00247-y

**Published:** 2021-02-22

**Authors:** Yu Liu, Risheng Huang, Deyao Xie, Xiaoming Lin, Liangcheng Zheng

**Affiliations:** 1 Department of Thoracic Surgery, The First Affiliated Hospital of Wenzhou Medical University, Wenzhou, China; 2 Department of Thoracic Surgery, Wenzhou Central Hospital, Wenzhou, China

**Keywords:** Cell cycle arrest, Cell growth, p21, ZNF674-AS1

## Abstract

**Background:**

ZNF674-AS1, a recently characterized long noncoding RNA, shows prognostic significance in hepatocellular carcinoma and glioma. However, the expression and function of ZNF674-AS1 in non-small cell lung cancer (NSCLC) are unclear.

**Methods:**

In this work, we investigated the expression of ZNF674-AS1 in 83 pairs of NSCLC specimens and adjacent noncancerous lung tissues. The clinical significance of ZNF674-AS1 in NSCLC was analyzed. The role of ZNF674-AS1 in NSCLC growth and cell cycle progression was explored.

**Results:**

Our data show that ZNF674-AS1 expression is decreased in NSCLC compared to normal tissues. ZNF674-AS1 downregulation is significantly correlated with advanced TNM stage and decreased overall survival of NSCLC patients. Overexpression of ZNF674-AS1 inhibits NSCLC cell proliferation, colony formation, and tumorigenesis, which is accompanied by a G0/G1 cell cycle arrest. Conversely, knockdown of ZNF674-AS1 enhances the proliferation and colony formation of NSCLC cells. Biochemically, ZNF674-AS1 overexpression increases the expression of p21 through downregulation of miR-423-3p. Knockdown of p21 or overexpression of miR-423-3p blocks ZNF674-AS1-mediated growth suppression and G0/G1 cell cycle arrest. In addition, ZNF674-AS1 expression is negatively correlated with miR-423-3p in NSCLC specimens.

**Conclusions:**

ZNF674-AS1 suppresses NSCLC growth by downregulating miR-423-3p and inducing p21. This work suggests the therapeutic potential of ZNF674-AS1 in the treatment of NSCLC.

## Background

Lung cancer is the leading cause of cancer-related deaths among males worldwide [1, 2]. Non-small cell lung cancer (NSCLC) accounts for over 80% of all lung cancer cases. Although advances have been made in therapeutic methods, the 5-year survival rate of patients with advanced NSCLC is less than 20% [3, 4]. Therefore, exploration of the molecular mechanisms governing NSCLC progression is important to develop effective therapies against this disease.

Long noncoding RNAs (lncRNAs) are a class of transcripts lacking protein-coding potential, with a length of over 200 nucleotides [5]. Accumulating evidence indicates that lncRNAs are aberrantly expressed in various cancer types and play a critical role in tumor progression [6, 7]. For example, lncRNA JPX is upregulated in metastatic lung cancer and shows the capacity to promote lung cancer growth and metastasis [6]. It has been suggested that lncRNAs regulate gene expression through interaction with microRNAs (miRNAs), which are small noncoding RNAs 22–24 nucleotides in length [8, 9]. Specially, lncRNAs may act as a sponge of miRNAs to release miRNA targets [10, 11]. Also, lncRNAs can suppress the expression of miRNAs [12].

Although several lncRNAs such as JPX [6], UCA1 [7], and JHDM1D-AS1 [13] have been reported to coordinate lung cancer progression, most aberrantly expressed lncRNAs in lung cancer remain uncharacterized. The lncRNA ZNF674 antisense RNA 1 (ZNF674-AS1) is downregulated in hepatocellular carcinoma (HCC) [14]. Moreover, ZNF674-AS1 downregulation is significantly correlated with distant metastasis, clinical stage, histopathologic grade, and poor prognosis in patients with HCC [14]. Another study analyzing the Chinese Glioma Genome Atlas microarray dataset revealed that ZNF674-AS1 serves as an unfavorable prognostic factor for glioma [15]. These preliminary results imply that ZNF674-AS1 may play a cell context-dependent role in tumor progression. However, the role of ZNF674-AS1 in NSCLC has not yet been clarified.

In the current study, we investigated the expression and clinical significance of ZNF674-AS1 in NSCLC. We performed gain- and loss-of-function experiments to determine the impact of ZNF674-AS1 on NSCLC aggressive phenotype. In addition, we identified the key miRNAs involved in the action of ZNF674-AS1 in NSCLC.

## Methods

### Patients and tissues

A total of 83 paired tumor samples and corresponding normal lung tissues were collected from NSCLC patients who underwent surgery at our hospital. Clinicopathological information of the patients is summarized in Additional file 1: Table S1. None was given any anticancer treatment before surgery. Tissue specimens were immediately frozen in liquid nitrogen and stored at − 80 °C.

### Cell culture

NSCLC cell lines A549, H1299, H358, and PC9 were cultured in Dulbecco’s Modified Eagle’s Medium (DMEM) supplemented with 10% fetal bovine serum (FBS; Invitrogen, Carlsbad, CA, USA) at 37 °C with 5% CO_2_ atmosphere. BEAS-2B cells were cultured in growth factor-supplemented medium (BEGM; Lonza, Walkersville, MD, USA). These cell lines were purchased from the Type Culture Collection of the Chinese Academy of Science (Shanghai, China). No mycoplasma infection was detected in the cell lines used in this study.

### Quantitative real-time PCR (qRT-PCR) analysis

Total RNA was extracted using TRIzol reagent (Invitrogen) and reverse-transcribed into cDNA using the Superscript III Reverse Transcriptase Kit (Invitrogen). qRT-PCR analysis of ZNF674-AS1 and p21 was performed using the following primers: ZNF674-AS1 forward, 5′-GCAGTGAATTACTGCTCATTC-3′ and reverse, 5′-GCCACAGATCAGGTGCTTCT-3′; p21 forward, 5′-GCCCAGTGGACAGCGAGCAG-3′ and reverse, 5′-GCCGGCGTTTGGAGTGGTAGA-3′ [16]. Glyceraldehyde-3-phosphate dehydrogenase (GAPDH) was used as an internal control. For quantification of mature miRNAs, total RNA was reverse-transcribed using the TaqMan miRNA Reverse Transcription kit (Applied Biosystems, Carlsbad, CA, USA) and amplified using the TaqMan miRNA assay system (Applied Biosystems). U6 was used as an endogenous control. The relative gene expression was determined by the 2^−ΔΔCT^ method [17].

### Plasmids, small interfering RNAs (siRNAs), miR-423-3p mimic, and anti-miR-423-3p

A fragment containing ZNF674-AS1 was amplified by PCR and cloned to the pcDNA3.1( +) expression vector. The p21-targeting siRNA (sip21) was purchased from Invitrogen. Two independent siRNAs targeting ZNF674-AS1 were synthesized by Sangon Biotechnology (Shanghai, China), with the target sequences as follows: ZNF674-AS1 siRNA#1, 5′-CCTAGATGGCTGTTGTTAT-3′, and ZNF674-AS1 siRNA#2, 5′-ATCTGATGTTAACAGTTGT-3′. miR-423-3p mimic was purchased from Sigma-Aldrich (St. Louis, MO, USA). Anti-miR-423-3p inhibitors were purchased from Thermo Fisher Scientific (Wilmington, MA, USA). Cell transfection was performed using Lipofectamine 3000 transfection reagent (Invitrogen), following the manufacturer’s instructions.

### MTT assay

Cells were plated in 96-well plates (5 × 10^3^ cells per well). After culturing for 24–72 h, cells were collected and assessed by the 3-(4,5-dimethylthiazol-2-yl)-2,5-diphenyl-2H-tetrazolium bromide (MTT) assay. In brief, MTT (0.5 mg/ml; Sigma-Aldrich) was added to each well and cultured at 37 °C for 4 h. The purple precipitates were dissolved in dimethyl sulfoxide (Sigma-Aldrich). Absorbance was measured at 490 nm.

### Colony formation assay

Colony formation assay was performed as described previously [18]. Cells were seeded onto 6-well plates (600 cells per well). After incubation for 10 days, cells were fixed and stained with crystal violet (Sigma-Aldrich). The number of colonies per well was counted.

### Animal studies

Male BALB/c nude mice, 5 weeks of age, were acclimated to the facility environment (12-h light/dark cycle, 23 ± 2 °C, and 50% humidity) for 1 week. Xenograft tumors were generated by injecting stably transfected A549 cells (2 × 10^6^) into the flanks of the mice. Tumor volume was measured every week. After 4 weeks mice were euthanatized, and tumors were weighed.

### Immunohistochemistry

Xenograft tumors were fixed and sectioned. The sections were deparaffinized, probed with anti-Ki-67 antibody (Sigma-Aldrich) in a humidified chamber, and then incubated with horseradish peroxidase (HRP)-conjugated secondary antibody. Signals were developed using 3,3′-diaminobenzidine solution (Sigma-Aldrich). The sections were counterstained with hematoxylin.

### Cell cycle analysis

For analysis of cell cycle progression, cells were fixed with 70% ethanol and stained with 50 μg/mL propidium iodide (PI) in the presence of 50 μg/mL RNase A (Sigma-Aldrich). Stained cells were analyzed by flow cytometry.

### Western blot analysis

Cells were lysed in ice-cold RIPA lysis buffer supplemented with a protease inhibitor cocktail (Sigma-Aldrich). Protein samples (30 μg/lane) were separated by sodium dodecyl sulfate polyacrylamide gel electrophoresis and transferred onto polyvinylidene fluoride membranes. The membranes were incubated overnight at 4 °C with primary antibodies recognizing p21 and GAPDH. These antibodies were purchased from Cell Signaling Technology (Danvers, MA, USA). The membranes were then incubated with secondary antibodies conjugated to HRP (Santa Cruz Biotechnology, Santa Cruz, CA, USA) for 1 h. Enhanced chemiluminescent reagents (Millipore, Billerica, MA, USA) were used to visualize bound antibodies.

### Statistical analysis

Data are expressed as mean ± standard deviation. Significant differences were analyzed by Student’s *t* test or one-way analysis of variance. The relationship of ZNF674-AS1 with clinicopathological parameters was analyzed using the chi-square test. Survival analysis was performed by the Kaplan–Meier method. Pearson correlation analysis was conducted to determine the correlation between ZNF674-AS1 and miR-423-3p. *P* < 0.05 was considered statistically significant.

## Results

### ZNF674-AS1 is downregulated and predicts poor prognosis in NSCLC

**Fig. 1:**
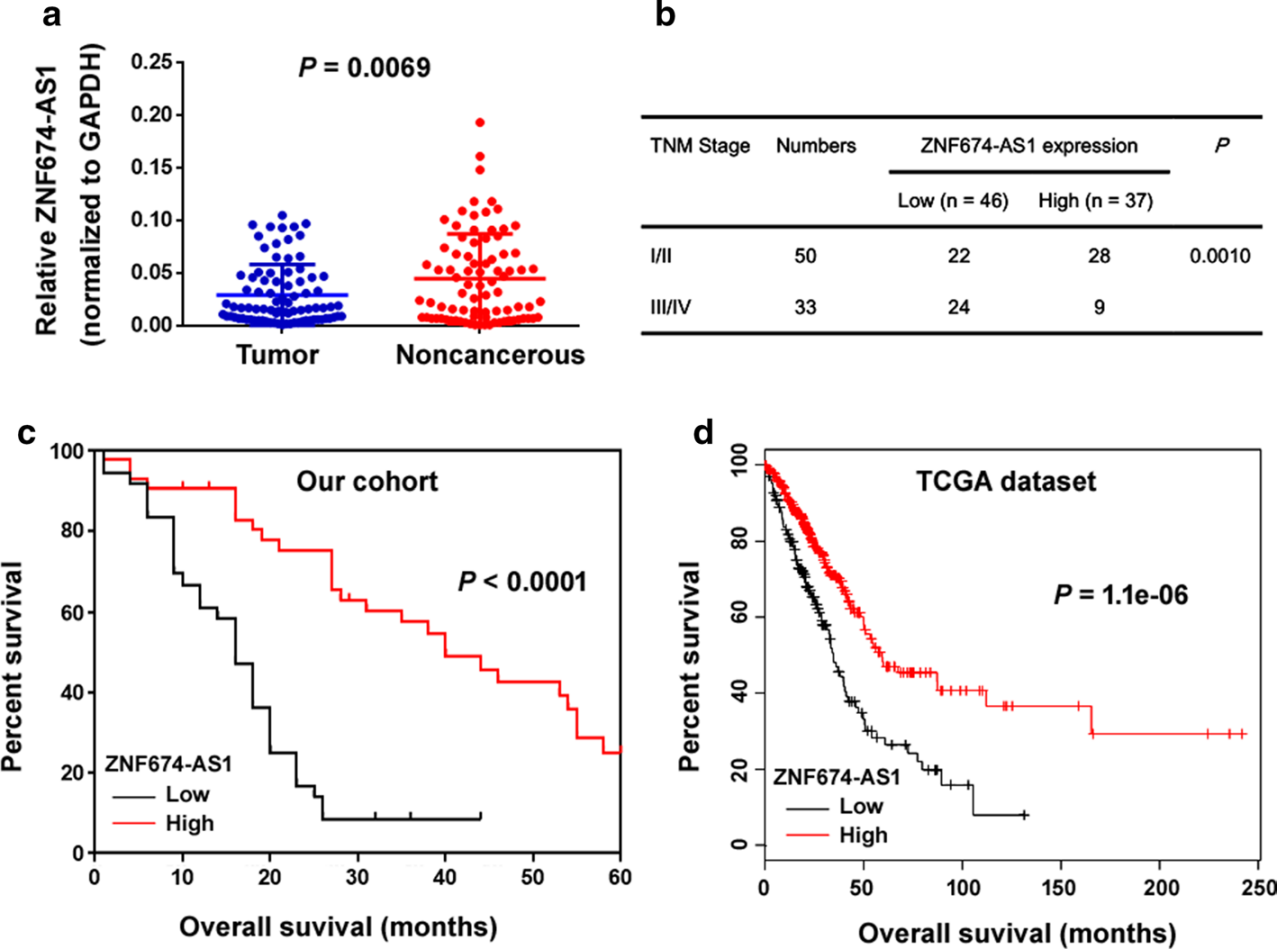
ZNF674-AS1 is downregulated and predicts poor prognosis in NSCLC. **a** Analysis of ZNF674-AS1 levels in 83 pairs of NSCLC specimens and adjacent noncancerous lung tissues. **b** Downregulation of ZNF674-AS1 was significantly associated with advanced TNM stage of NSCLC. **c** Kaplan–Meier analysis showed that reduced ZNF674-AS1 expression was associated with shorter overall survival of NSCLC patients. **d** Based on the lung adenocarcinoma TCGA dataset included in KM Plotter, ZNF674-AS1 expression was associated with decreased overall survival

To explore the expression of ZNF674-AS1 in NSCLC, we analyzed the expression of ZNF674-AS1 in 83 pairs of NSCLC specimens and adjacent noncancerous lung tissues. The results showed that ZNF674-AS1 expression was significantly decreased in NSCLC relative to normal tissues (*P* = 0.0069; Fig. 1a). We then analyzed ZNF674-AS1 levels in tumors grouped by TNM staging. Notably, downregulation of ZNF674-AS1 was significantly correlated with advanced TNM stage (*P* = 0.0010; Fig. 1b). Consistently, Kaplan–Meier analysis indicated that NSCLC patients with low ZNF674-AS1 levels had a shorter overall survival than those with high ZNF674-AS1 levels (*P* < 0.0001; Fig. 1c). In addition, analysis of ZNF674-AS1 expression in 504 lung adenocarcinoma specimens using KM Plotter (http://kmplot.com/analysis/) confirmed that low ZNF674-AS1 expression was associated with decreased overall survival (Fig. 1d). These 504 samples are derived from The Cancer Genome Atlas. These data suggest that ZNF674-AS1 downregulation may contribute to NSCLC progression.

### ZNF674-AS1 suppresses NSCLC cell proliferation and colony formation

**Fig. 2:**
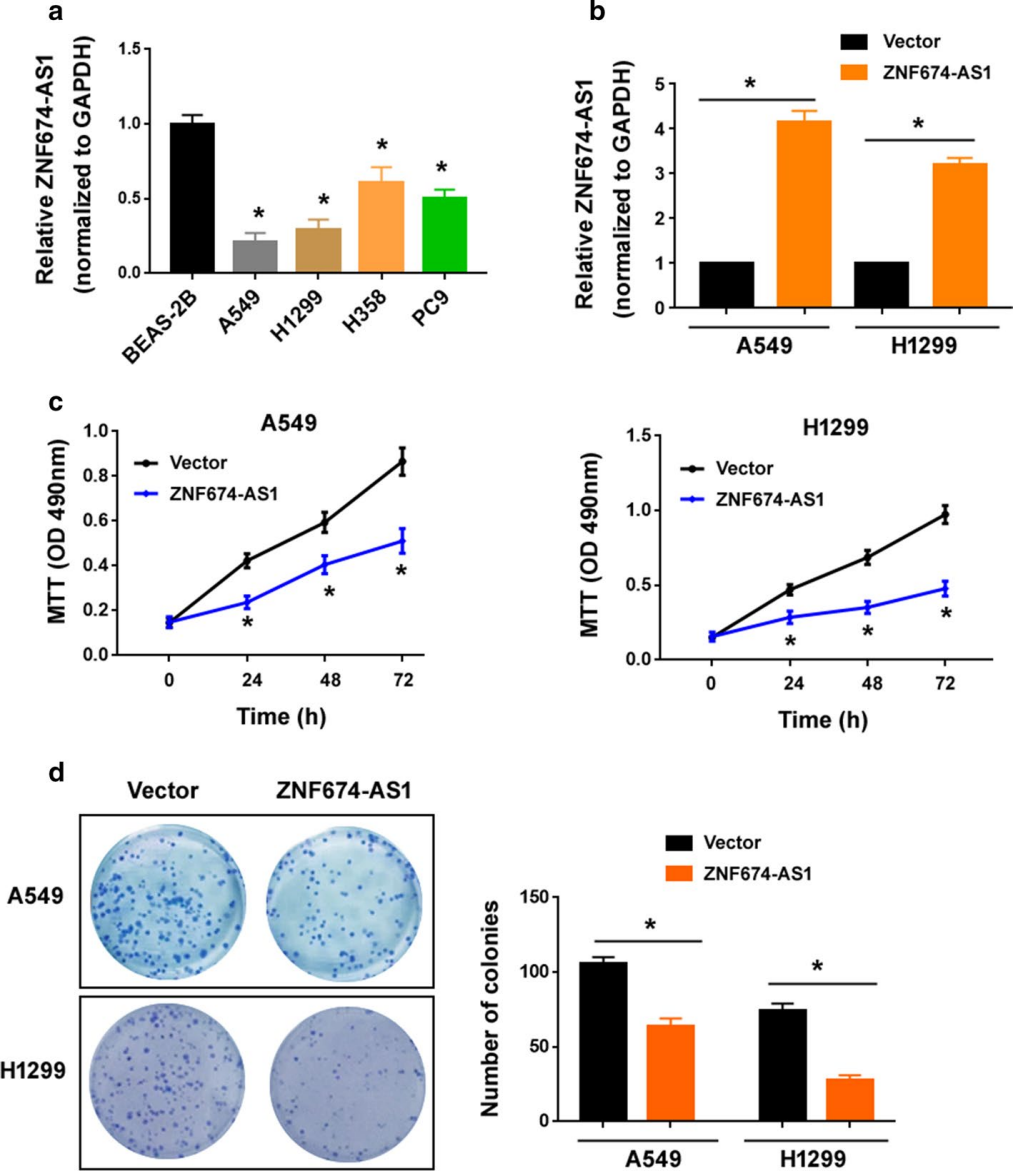
ZNF674-AS1 suppresses NSCLC cell proliferation and colony formation. **a** Expression of ZNF674-AS1 in indicated cell lines. ^*^P < 0.05 compared to BEAS-2B cells. **b** Overexpression of ZNF674-AS1 in both A549 and H1299 cells. **c** As determined by MTT assay, NSCLC cell growth was suppressed by overexpression of ZNF674-AS1. **d** Colony formation assay showed that ZNF674-AS1 suppressed colony formation capacity of NSCLC cells. ^*^P < 0.05 vs. Vector

Consistent with the clinical findings, ZNF674-AS1 expression was downregulated in the NSCLC cell lines tested compared to BEAS-2B bronchial epithelial cells (Fig. 2a). To determine the role of ZNF674-AS1 in NSCLC cell growth and invasion, ZNF674-AS1 was ectopically overexpressed in both A549 and H1299 cells (Fig. 2b), which had low levels of endogenous ZNF674-AS1. The growth of NSCLC cells was significantly suppressed by overexpression of ZNF674-AS1 (Fig. 2c). Moreover, ZNF674-AS1-mediated growth suppression was confirmed in colony formation assays (Fig. 2d). However, ZNF674-AS1 overexpression had no impact on the invasion ability of NSCLC cells (Additional file 1: Figure S1).

**Fig. 3:**
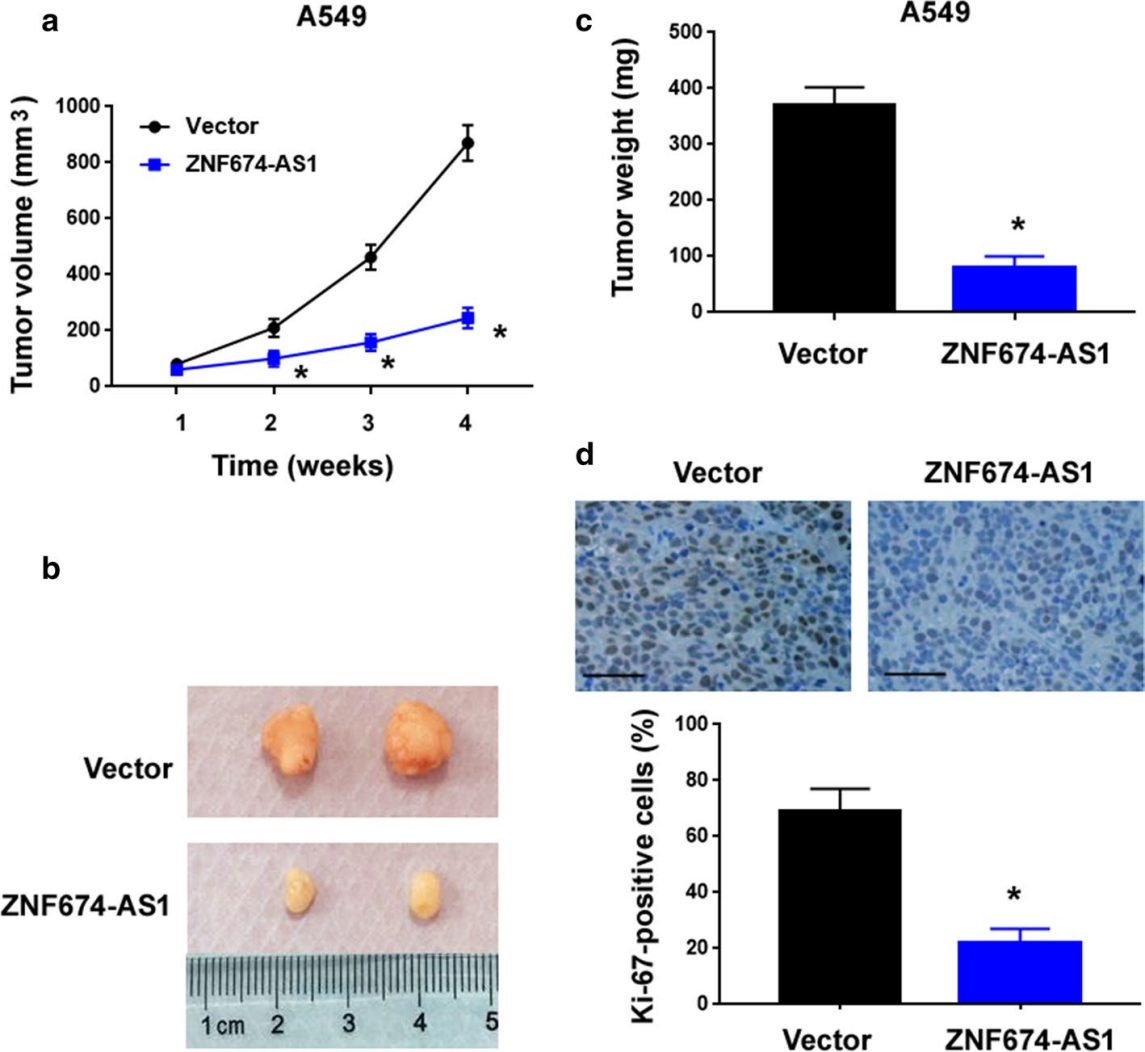
ZNF674-AS1 blocks tumorigenesis of A549 cells in vivo. **a** Assessment of the growth of xenograft tumors formed by ZNF674-AS1-overexpressing and control A549 cells. **b** Photographs of 2 representative xenograft tumors from each group. **c** Tumor weight was measured 4 weeks after cell inoculation. **d** Ki-67 immunostaining performed in xenograft tumors. Scale bar = 100 μm. Quantitative results of Ki-67 staining are shown in bottom panel. ^*^P < 0.05 vs. Vector

We further investigated the effect of ZNF674-AS1 on A549 cell tumorigenesis in a nude mouse model. Tumor volume measurements demonstrated that ZNF674-AS1-overexpressing A549 xenografts were significantly smaller than control xenografts (Fig. 3a). At 4 weeks after cell inoculation, tumor weight in the ZNF674-AS1-overexpressing group was ~ fourfold lower than that in the control group (Figs. 3b and c). Histological analysis confirmed that there were fewer ki-67-positive proliferating cells in ZNF674-AS1-overexpressing xenografts than in control xenografts (Fig. 3d). Collectively, these data indicate that ZNF674-AS1 can exert growth-suppressing activity in NSCLC.

### Knockdown of ZNF674-AS1 accelerates NSCLC cell growth

**Fig. 4:**
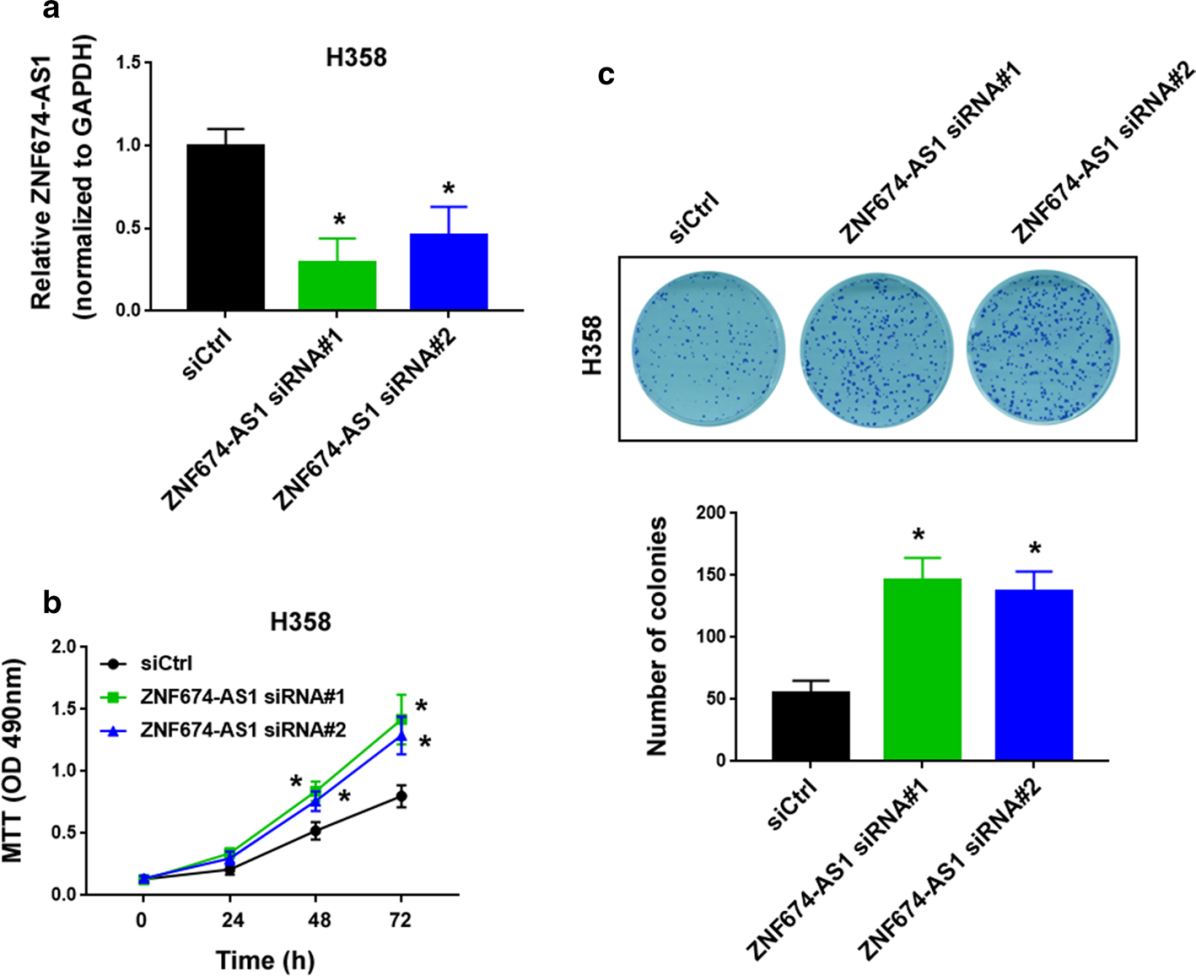
Knockdown of ZNF674-AS1 accelerates NSCLC cell growth and colony formation. **a** The level of ZNF674-AS1 transcript was analyzed in H358 cells transfected with control siRNA (siCtrl) or ZNF674-AS1-targeting siRNAs. **b** MTT assay showed the induction of proliferation in ZNF674-AS1-depleted H358 cells. **c** Assessment of colony formation in H358 cells transfected with indicated siRNAs. ^*^P < 0.05 vs. siCtrl

To gain further insight into the role of ZNF674-AS1 in NSCLC, we performed ZNF674-AS1 knockdown experiments in H358 cells using siRNA technology. As measured by qPCR analysis, transfection with ZNF674-AS1 siRNA led to a reduction of ZNF674-AS1 abundance in H358 cells by 60–70% (Fig. 4a). The downregulation of ZNF674-AS1 increased the proliferation of H358 cells, as revealed by the MTT assay (Fig. 4b). Moreover, colony formation was enhanced in ZNF674-AS1-depleted H358 cells (Fig. 4c). Hence, ZNF674-AS1 exerts anti-proliferative effects on NSCLC cells.

### ZNF674-AS1 causes a G0/G1 cell cycle arrest through upregulation of p21

**Fig. 5:**
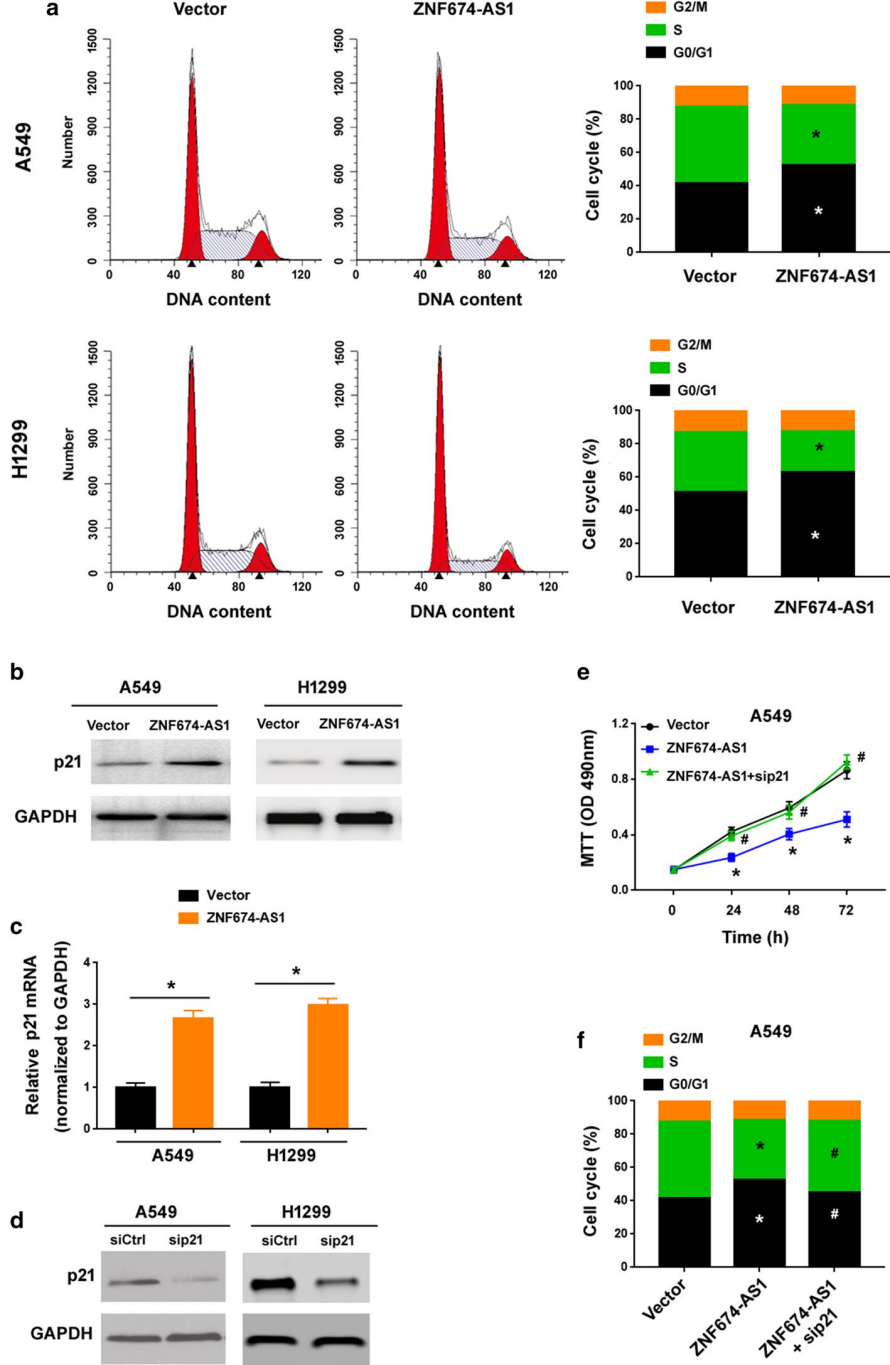
ZNF674-AS1 causes a G0/G1 cell cycle arrest through upregulation of p21. **a** Flow cytometric analysis showed that overexpression of ZNF674-AS1 arrested NSCLC cells at the G0/G1 phase. ^*^P < 0.05 compared to vector-transfected cells. **b** Western blot analysis of indicated proteins. **c** ZNF674-AS1 overexpression increased the levels of p21 mRNA in NSCLC cells. ^*^P < 0.05. **d** Knockdown of p21 in NSCLC cells by transfection of siRNAs. siCtrl: control siRNA; sip21: p21-targeting siRNA. **e** MTT assay showed that p21 knockdown rescued ZNF674-AS1-mediated growth of A549 cells. **f** Cell cycle analysis conducted in A549 cells transfected with indicated constructs. ^*^P < 0.05 compared to vector-transfected cells; ^#^*P* < 0.05 compared to cells transfected with ZNF674-AS1 alone

Next, we asked whether ZNF674-AS1-mediated growth suppression was ascribed to induction of cell cycle arrest. Flow cytometric analysis of cell cycle distribution revealed that overexpression of ZNF674-AS1 increased the number of cells in the G1 phase and decreased the number of cells in the S phase (Fig. 5a), indicating a partial cell cycle arrest at the G0/G1 phase. We further examined the expression of a number of cell cycle-related genes. The results demonstrated that overexpression of ZNF674-AS1 led to a marked increase of p21 protein in both A549 and H1299 cells (Fig. 5b). Other cell cycle-related genes including cyclin D1, p27, p16 and Ki67 remained unaffected (data not shown). Moreover, the level of p21 mRNA was elevated in ZNF674-AS1-overexpressing NSCLC cells (Fig. 5c). Therefore, we speculated that p21 was involved in the growth-suppressing activity of ZNF674-AS1. To validate the hypothesis, we performed p21 knockdown experiments (Fig. 5d). As shown in Fig. 5e and f, p21 knockdown rescued ZNF674-AS1-mediated growth suppression and G0/G1 cell cycle arrest. The results suggest that upregulation of p21 at least partially contributes to the ZNF674-AS1 phenotypes observed.

### ZNF674-AS1 antagonizes miR-423-3p to derepress p21

**Fig. 6:**
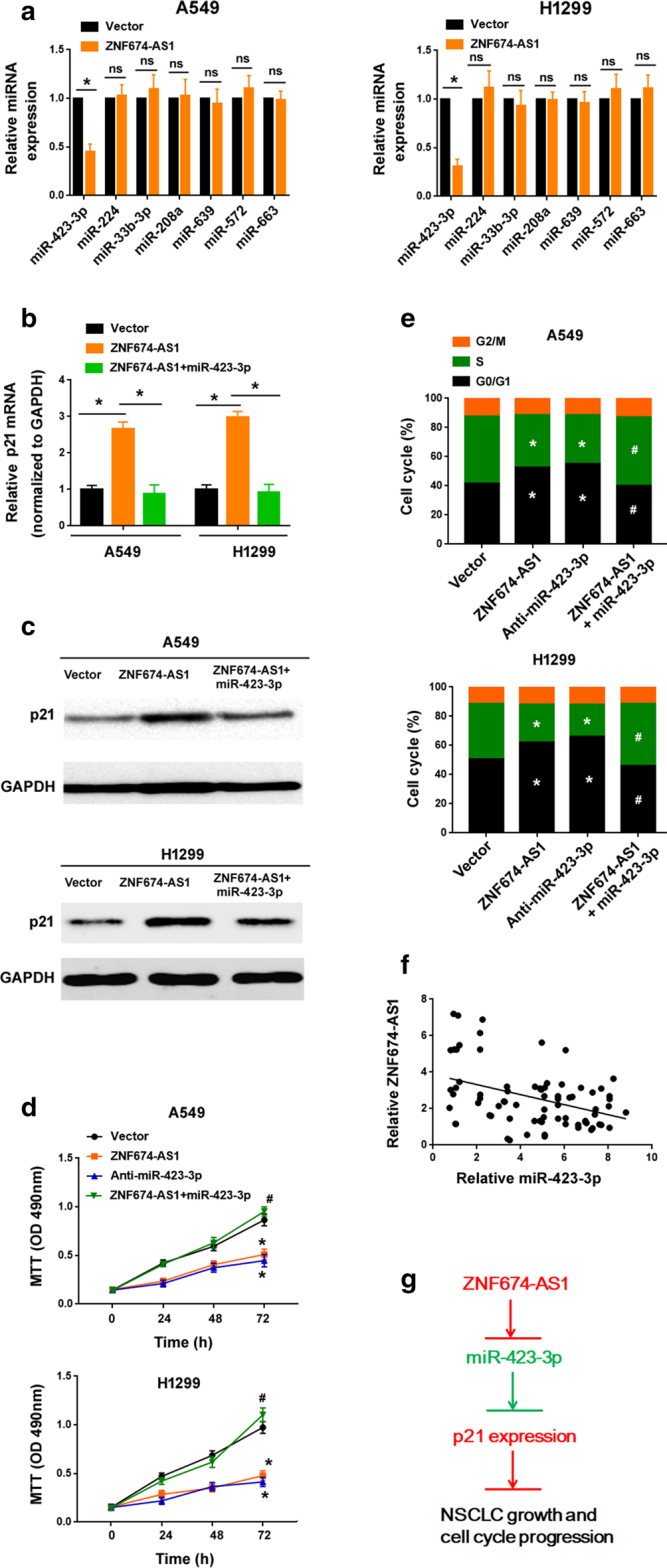
ZNF674-AS1 antagonizes miR-423-3p to derepress p21. **a** Analysis of candidate miRNAs in NSCLC cells transfected with vector or ZNF674-AS1. ^*^P < 0.05. ns: no significance. **b** Analysis of p21 mRNA levels after transfection with indicated constructs. ^*^P < 0.05. **c** Western blot analysis of p21 protein levels in NSCLC cells treated as in (**b**). **d** MTT assay showed that ZNF674-AS1-mediated growth suppression was reversed by miR-423-3p. **e** Cell cycle analysis conducted in NSCLC cells transfected with indicated constructs. ^*^P < 0.05 compared to vector-transfected cells; ^#^*P* < 0.05 compared to cells transfected with ZNF674-AS1 alone. **f** An inverse correlation was detected between expression of ZNF674-AS1 and miR-423-3p in NSCLC specimens (n = 83). **g** Schematic model of ZNF674-AS1/miR-423-3p/p21 pathway

Due to the interplay between lncRNAs and miRNAs in the regulation of gene expression [6, 7], we asked whether ZNF674-AS1 could promote p21 through interaction with specific miRNAs. To address this issue, we examined the effect of ZNF674-AS1 overexpression on the expression of multiple candidate miRNAs [19–25], which have shown the ability to repress p21. Interestingly, overexpression of ZNF674-AS1 caused a decline in the level of miR-423-3p but not miR-224, miR-33b-3p, miR-208a, miR-639, miR-572, or miR-663 (Fig. 6a). Overexpression of miR-423-3p attenuated ZNF674-AS1-dependent induction of p21 (Fig. 6b, c). Moreover, ZNF674-AS1-mediated growth suppression and cell cycle arrest was reversed by miR-423-3p overexpression (Fig. 6d, e). Depletion of miR-423-3p (Additional file 1: Figure S2) phenocopied ZNF674-AS1 overexpression in NSCLC cells (Fig. 6d, e). In addition, there was an inverse correlation between the expression of ZNF674-AS1 and miR-423-3p in NSCLC specimens (r = − 0.407, *P* = 0.0002; Fig. 6f).

## Discussion

Previous studies have identified a number of lncRNAs that are dysregulated in NSCLC and serve as potential prognostic biomarkers for this malignancy [13, 26]. For instance, upregulation of lncRNA SNHG3 by E2F1 is associated with a low overall survival rate of patients with NSCLC [26]. In this study, we report a novel prognostic factor for NSCLC. We show that ZNF674-AS1 is downregulated in NSCLC relative to corresponding normal lung tissues. The downregulation of ZNF674-AS1 is associated with advanced TNM stage and reduced overall survival of NSCLC patients. The potential of ZNF674-AS1 as a prognostic biomarker has also been described in HCC [14] and glioma [15].

Biochemically, ZNF674-AS1 overexpression inhibits the proliferation and tumorigenesis of NSCLC cells. In contrast, the invasive capacity of NSCLC cells is not affected by ZNF674-AS1 overexpression. Our data provide the first evidence for the role of ZNF674-AS1 in modulating tumor progression. The tumor-suppressive activity of ZNF674-AS1 may provide an explanation for the clinical relationship between ZNF674-AS1 downregulation and reduced overall survival of NSCLC patients. Multiple lncRNAs have exhibited the capacity to regulate cell cycle progression [27, 28]. For instance, knockdown of lncRNA EPIC1 leads to growth suppression and G0/G1 cell cycle arrest in pancreatic cancer cells [27]. The lncRNA EPIC1 has been shown to promote cell cycle progression in cancer cells through interaction with MYC [28]. Our data indicate ZNF674-AS1 as a negative regulator of cell cycle progression in NSCLC cells. In particular, ectopic expression of ZNF674-AS1 arrests NSCLC cells at the G0/G1 phase. Therefore, ZNF674-AS1 inhibits NSCLC growth, at least partially through inducing G0/G1 cell cycle arrest.

Mechanistically, ZNF674-AS1 selectively stimulates the expression of p21 in NSCLC cells. p21 belongs to the Cip and Kip family of CDK inhibitors [29]. It exerts biological activities primarily by inhibiting the kinase activity of CDKs [30, 31]. Induction of p21 has been shown to impair the G1/S cell cycle transition in NSCLC cells by inhibiting the activation of CDK2 complexes [30]. Interestingly, we show that knockdown of p21 prevents growth suppression of NSCLC cells induced by ZNF674-AS1. Therefore, ZNF674-AS1-mediated antiproliferative activity in NSCLC cells depends on the upregulation of p21.

It has been suggested that lncRNAs can regulate gene expression through interaction with miRNAs [6, 7]. For instance, lncRNA XIST is capable of inducing p21 through interaction with miR-106b-5p in renal cell carcinoma [32]. Similarly, Nrf2-lncRNA can promote the expression of p21 by sponging miR-128 and miR-224 [33]. Here, we show that ZNF674-AS1 overexpression suppresses the expression of miR-423-3p in NSCLC cells. miR-423-3p has been shown to promote lung cancer proliferation and invasion [34]. Previous studies have reported p21 as a direct target of miR-423-3p [23, 35]. Overexpression of miR-423-3p was found to inhibit p21 expression in HCC [23] and colorectal cancer [35]. Consistently, our data indicate that restoration of miR-423-3p blocks the induction of p21 by ZNF674-AS1 in NSCLC cells. Biologically, ZNF674-AS1-mediated anticancer activity is counteracted by miR-423-3p overexpression. Clinically, ZNF674-AS1 expression is negatively correlated with miR-423-3p in NSCLC tissues. Considering all the evidence, we suggest that ZNF674-AS1 exerts its suppressive activity against NSCLC through inhibition of miR-423-3p and subsequent derepression of p21 (Fig. 6g). However, future work is needed to identify the direct mediators of ZNF674-AS1 suppressive activity in NSCLC.

## Conclusion

We identify ZNF674-AS1 as a new growth suppressor in NSCLC. Induction of p21 due to miR-423-3p downregulation is responsible for ZNF674-AS1-mediated growth suppression. Our data suggest that restoration of ZNF674-AS1 represents a potential therapeutic strategy to treat NSCLC.

## Supplementary Information

**Additional file 1: Table S1**. Clinicopathological features of NSCLC patients (n = 83). **Figure S1.** Effect of ZNF674-AS1 overexpression on the invasion of NSCLC cells. (A) Representative images of Transwell invasion assay. (B) Quantification results of Transwell invasion assay. ns: no significance. **Figure S2.** Quantification of miR-423-3p expression in A549 and H1299 cells transfected with negative control or anti-miR-423-3p by real-time PCR analysis.

## Data Availability

The datasets generated during and/or analyzed during the current study are available from the corresponding author on reasonable request.

## Abbreviations

lncRNA: Long noncoding RNA
miRNA: MicroRNA
NSCLC: Non-small cell lung cancer
PI: Propidium iodide
qRT-PCR: Quantitative real-time PCR
