# Supplementary material for: ZNF674-AS1 antagonizes miR-423-3p to induce G0/G1 cell cycle arrest in non-small cell lung cancer cells
Source: Cell Mol Biol Lett. 2021 Feb 22;26:6. doi: 10.1186/s11658-021-00247-y (PMC7901084; doi:10.1186/s11658-021-00247-y)
Supplement: Supplementary file 1 — Additional file 1: Table S1. Clinicopathological features of NSCLC patients (n = 83). Figure S1. Effect of ZNF674-AS1 overexpression on the invasion of NSCLC cells. (A) Representative images of Transwell invasion assay. (B) Quantification results of Transwell invasion assay. ns: no significance. Figure S2. Quantification of miR-423-3p expression in A549 and H1299 cells transfected with negative control or anti-miR-423-3p by real-time PCR analysis. [file 11658_2021_247_MOESM1_ESM.pdf]

Additional Table S1 Clinicopathological features of NSCLC patients (n = 83)

**Variable**	**n**
Age	
<65	38
≥65	45
Sex	
Male	55
Female	28
TNM stage	
I-II	50
III-IV	33
Histological subtype	
Squamous cell carcinoma	22
Adenocarcinoma	61
Lymph node metastasis	
Negative	47
Positive	36

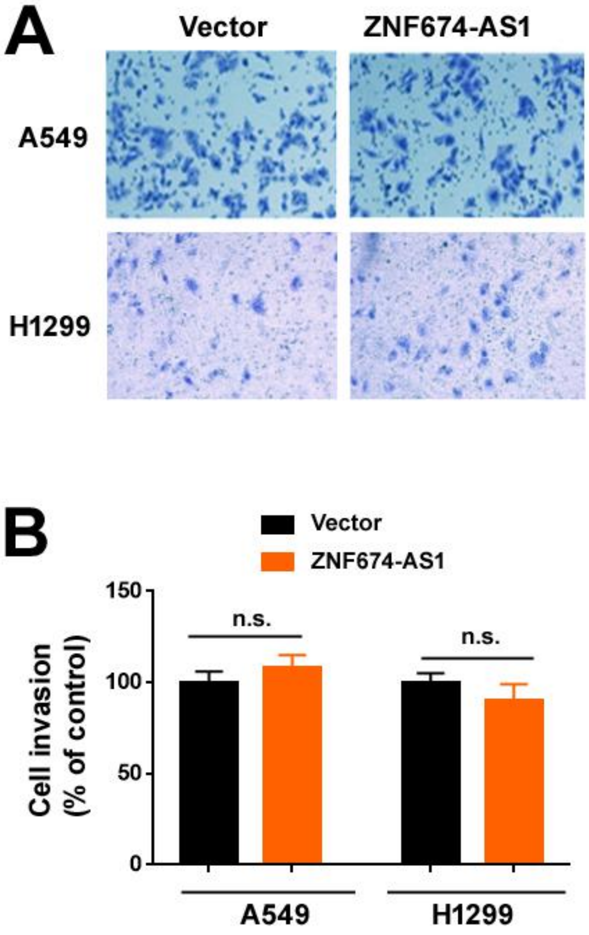

Additional Figure S1. Effect of ZNF674-AS1 overexpression on the invasion of NSCLC cells. (A) Representative images of Transwell invasion assay. (B) Quantification results of Transwell invasion assay. ns: no significance.

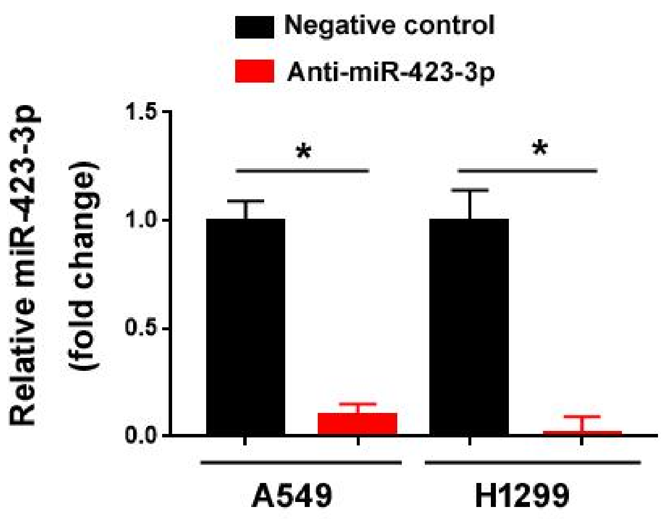

Additional Figure S2. Quantification of miR-423-3p expression in A549 and H1299 cells transfected with negative control or anti-miR-423-3p by real-time PCR analysis.

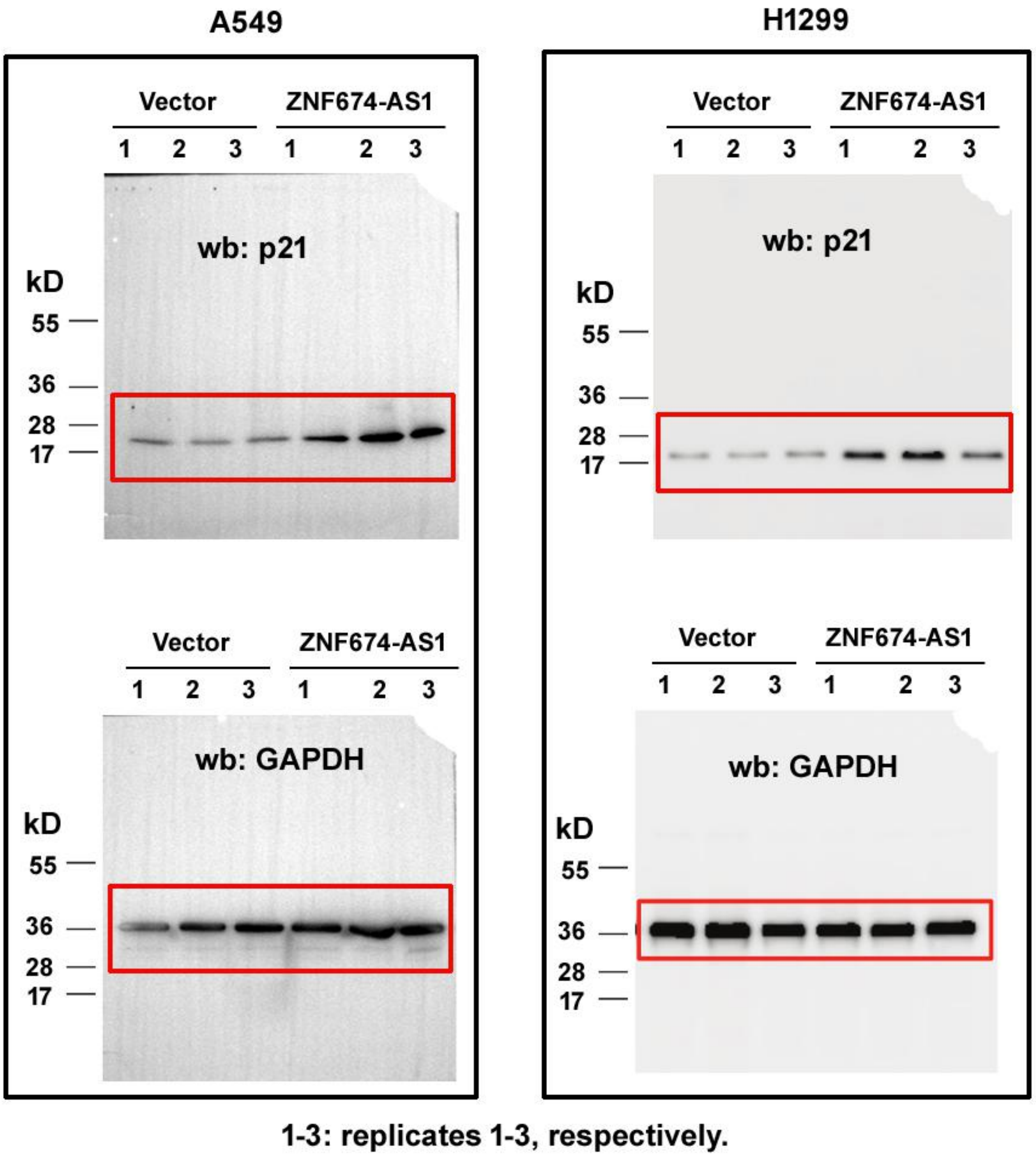

Original Western blot images for Figure 5B.

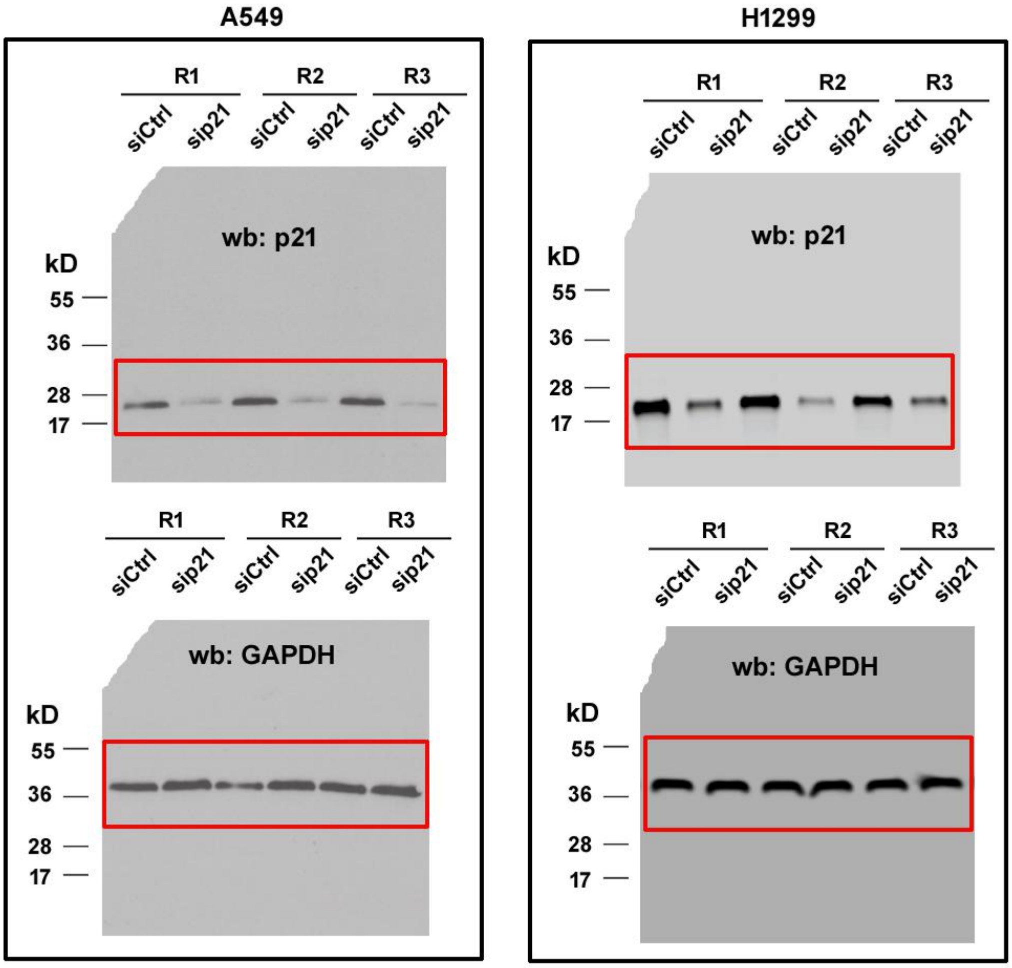

R1, R2, and R3: replicate 1, replicate 2, and replicate 3, respectively.

Original Western blot images for Figure 5D.

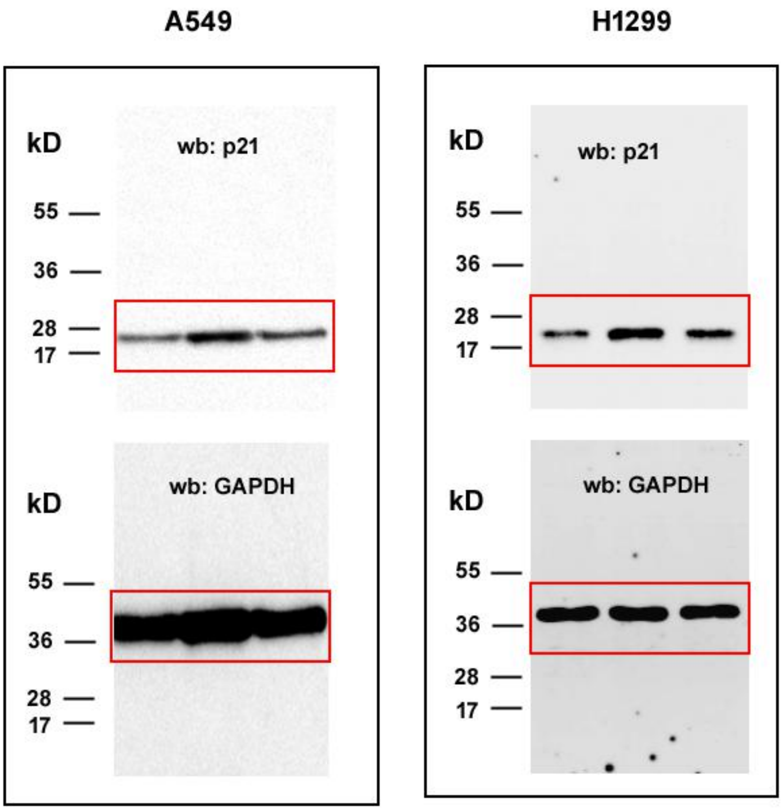

Original Western blot images for Figure 6C.
